# High-resolution sleep fragmentation assessment in narcolepsy type 1 and their non-narcoleptic siblings: a 5-s mini-epoch study

**DOI:** 10.1093/sleep/zsag015

**Published:** 2026-01-28

**Authors:** Louise Frøstrup Follin, Rannveig Viste, Janita Vevelstad, Kristin Langdalen, Berit Hjelde Hansen, Ragnhild Kristine Berling Grande, Tobias Kaufmann, Julie Anja Engelhard Christensen, Alexander Neergaard Zahid, Marte Kathrine Viken, Hilde T Juvodden, Stine Knudsen-Heier

**Affiliations:** Norwegian Centre of Expertise for Neurodevelopmental Disorders and Hypersomnias (NevSom), Department of Rare Disorders, Oslo University Hospital, Oslo, Norway; Institute of Clinical Medicine, University of Oslo, Oslo, Norway; Norwegian Centre of Expertise for Neurodevelopmental Disorders and Hypersomnias (NevSom), Department of Rare Disorders, Oslo University Hospital, Oslo, Norway; Norwegian Centre of Expertise for Neurodevelopmental Disorders and Hypersomnias (NevSom), Department of Rare Disorders, Oslo University Hospital, Oslo, Norway; Norwegian Centre of Expertise for Neurodevelopmental Disorders and Hypersomnias (NevSom), Department of Rare Disorders, Oslo University Hospital, Oslo, Norway; Institute of Clinical Medicine, University of Oslo, Oslo, Norway; Norwegian Centre of Expertise for Neurodevelopmental Disorders and Hypersomnias (NevSom), Department of Rare Disorders, Oslo University Hospital, Oslo, Norway; Norwegian Centre of Expertise for Neurodevelopmental Disorders and Hypersomnias (NevSom), Department of Rare Disorders, Oslo University Hospital, Oslo, Norway; Centre for Precision Psychiatry, Institute of Clinical Medicine, University of Oslo, Oslo, Norway; Department of Psychiatry and Psychotherapy, University of Tübingen, Tübingen, Germany; German Center for Mental Health (DZPG), Partner site Tübingen, Tübingen, Germany; Norwegian Centre of Expertise for Neurodevelopmental Disorders and Hypersomnias (NevSom), Department of Rare Disorders, Oslo University Hospital, Oslo, Norway; Primary Packaging Development, Novo Nordisk, Hillerød, Denmark; AI Center of Excellence, WS Audiology, Lynge, Denmark; Department of Applied Mathematics and Computer Science, Technical University of Denmark, Kgs. Lyngby, Denmark; Department of Immunology, University of Oslo and Oslo University Hospital, Oslo, Norway; Department of Medical Genetics, University of Oslo and Oslo University Hospital, Oslo, Norway; Norwegian Centre of Expertise for Neurodevelopmental Disorders and Hypersomnias (NevSom), Department of Rare Disorders, Oslo University Hospital, Oslo, Norway; Norwegian Centre of Expertise for Neurodevelopmental Disorders and Hypersomnias (NevSom), Department of Rare Disorders, Oslo University Hospital, Oslo, Norway; Institute of Clinical Medicine, University of Oslo, Oslo, Norway

**Keywords:** narcolepsy type 1, polysomnography, sleep fragmentation, mini-epochs, split-night analysis, U-sleep

## Abstract

**Study Objectives:**

Narcolepsy type 1 (NT1) is characterized by fragmented sleep, yet brief transitions may be concealed in traditional 30-s epochs. We therefore aimed to assess sleep fragmentation in NT1 patients and their non-narcoleptic siblings using 5-s mini-epochs.

**Methods:**

We analyzed sleep stages from human-scored 30-s epochs and automatically (U-Sleep) scored 5-s mini-epochs in polysomnographies from 125 NT1 patients and their 100 non-narcoleptic siblings. Sleep fragmentation was quantified using transition indices and probabilities in epochs and mini-epochs, furthermore, stratified by 1st and 2nd night-half. Predictors (H1N1-vaccination, CSF hypocretin-1 deficiency severity (low [40–150 pg/mL]; undetectable [<40 pg/mL]), HLA-DQB1^*^06:02-positivity, narcolepsy core symptom severity) for sleep transitions were explored.

**Results:**

NT1 patients had significantly higher all-stages and sleep–wake transition indices compared to siblings in epochs and mini-epochs. Both epoch and mini-epoch transition indices varied significantly from 1st to 2nd night-half. Only in mini-epochs, sleep–wake fragmentation was (a) higher in patients with severe core symptoms, and (b) increased in the 2nd night-half in NT1 patients with severe hypocretin-1 deficiency (<40 pg/mL) and/or severe core symptoms.

**Conclusions:**

Our findings suggest that sleep fragmentation, a core feature of NT1, is associated with disease severity and hypocretin deficiency severity when high-resolution sleep stages are analyzed. Five-second sleep staging and split-night analyses enhance detection of sleep instability and reveal new specific patterns and clinical associations. Automated mini-epoch analyses may improve future NT1 phenotyping and possibly its borderland by supplementing with clinically relevant high-resolution features otherwise hidden in traditional full-night 30-s epoch analyses.

**Statement of Significance**

This study demonstrates that high-resolution 5-s sleep staging provides more detailed sleep characterization than epochs and additionally uncovers clinically relevant sleep fragmentation patterns in narcolepsy type 1 which remain hidden in traditional 30-s analyses. By combining 5-s mini-epoch scoring with split-night analyses, we identified specific associations between disease severity and increased sleep–wake fragmentation. In mini-epochs, but not in epochs, sleep–wake fragmentation was significantly higher in patients with all core narcolepsy symptoms and increased significantly from the 1^st^ to the 2^nd^ night-half in patients with undetectable hypocretin-1 levels (<40 pg/mL) and/or all core narcolepsy symptoms. These findings highlight the clinical value of high-resolution, temporally sensitive sleep stage analyses for detailed phenotyping and detection of future disease biomarkers.

## Graphical Abstract

**Graphical Abstract:**
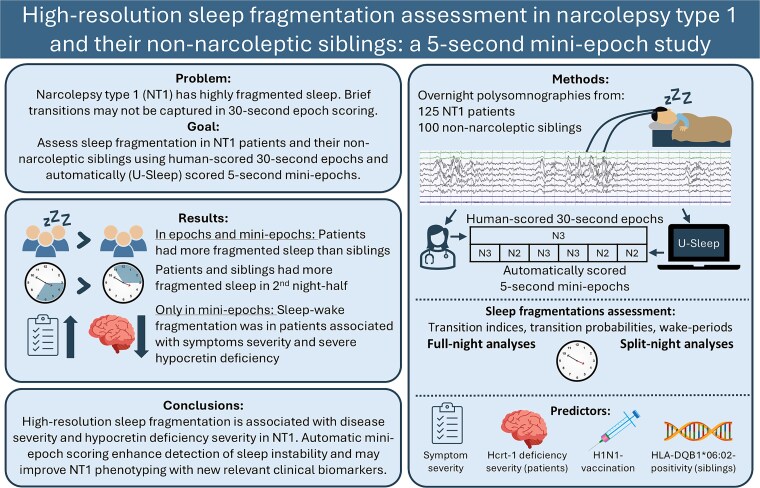

## Introduction

Narcolepsy type 1 (NT1) is a chronic sleep disorder characterized by excessive daytime sleepiness (EDS), cataplexy (a sudden loss of muscle tone triggered by emotions), hypnagogic hallucinations (HH), and sleep paralysis (SP) [1, 2]. In addition, NT1 is associated with early appearing rapid eye movement (REM) sleep (sleep onset REM periods [SOREMPs]) and pronounced sleep fragmentation, including frequent transitions between sleep stages and multiple awakenings [3, 4]. This instability of sleep–wake and REM sleep regulation has, in animal models and human NT1 studies, been linked to deficient signaling/neuron loss of the brain’s hypocretin (orexin) neurons in the lateral hypothalamus [5–12]. In humans, the strong association to the HLA-DQB1^*^06:02 allele [13, 14] supports autoimmune mechanisms for neuronal loss/dysfunction [15, 16]. Airway infections but also immunization have been suggested as potential triggers for NT1 development in HLA-predisposed individuals [17–20]. Specifically, in several European countries, including Norway, a substantial rise in NT1 incidence occurred after mass-vaccination with the H1N1-influenza vaccine Pandemrix in 2009–2010 [21–29].

Nighttime sleep fragmentation, defined by frequent transitions between sleep stages and sleep–wake instability, is a well-established feature of NT1 [3, 4]. In addition, NT1 patients also have more nocturnal wake-periods of variable duration, including both short and long segments of wakefulness [3, 4, 30]. Though the quantification of sleep fragmentation is not clinically standardized [3], it is typically based on polysomnographic recordings scored in 30-s epochs, in line with the current clinical American Academy of Sleep Medicine (AASM) guideline [31]. However, it is a well-known challenge that 30-s epochs can contain features from more than a single sleep stage [32–37]. Hence, conventional epoch scoring may conceal brief sleep stage transitions or short wake-periods, i.e. possibly also conceal significant polysomnography (PSG) biomarkers/signatures which could be particularly relevant in disorders with unstable sleep architecture like NT1.

Advances in automatic sleep staging have enabled high-resolution sleep stage scoring, allowing sleep stages to be scored in shorter intervals than the conventional 30-s epochs [35–38]. The problem has though in general been, that although such automatic classifiers score sleep very consistent and precise, they have originally only been trained to recognize human scored 30-s epochs as the “true” stages, i.e. it has been difficult to interpret the possible clinical relevance of their high-resolution assessments. Recently, our group therefore re-trained and validated the deep learning-based sleep stage classifier U-Sleep [38] against human scored 5-s mini-epochs for more accurate automatic scoring of 5-s mini-epochs [39]. With this approach, we achieved that the optimized U-Sleep model’s automated scorings of 5-s mini-epochs reached an agreement equally high as in the previously published automated 30-s sleep scoring studies [36, 38, 40–43] and additionally also equally high as inter-rater variability between human scorers of 30-s epochs [40, 44, 45]. Furthermore, we confirmed the findings of our previous smaller study, where we showed that 5-s mini-epoch scoring captures more stage transitions than conventional 30-s epoch scoring [46], offering the potential to better characterize sleep fragmentation.

Based on our own clinical experience when sleep scoring, nocturnal sleep in NT1 is often more fragmented toward the end of the night, possibly because the patients’ high sleep pressure at sleep onset “overcomes” the inherent sleep instability resulting in deeper and more consolidated early-night sleep, while fragmentation later in the night increases in parallel with a decreasing sleep pressure. This is also supported by previous NT1 publications of daytime sleep restriction and known patient strategies of skipping daytime/evening naps to try to reduce bothersome nightly sleep disruption [1, 47].

While sleep fragmentation in NT1 has previously been studied, the majority of these were based on 30-s epoch scorings [33, 48–58]. We therefore aimed to analyze whether 5-s sleep staging of PSGs, using our optimized re-trained version of U-Sleep [39], adds new insight about sleep instability in NT1. Furthermore, we aim to explore if disease severity (CSF hcrt-1 loss severity (undetectable vs. low but detectable levels); severity of the NT1 core symptoms [EDS, cataplexy, HH, SP]), and the known risk factor H1N1-vaccination with Pandemrix is associated with sleep fragmentation severity in NT1. Considering the increased risk of NT1 among first-degree relatives [59, 60] and findings suggesting a potential phenotype spectrum within this group [60–62], we also explore whether known risk factors (H1N1-vaccination, HLA-DQB1^*^06:02 positivity) or having at least one of the NT1 core symptoms (EDS, cataplexy-like episodes, HH, or SP) are associated with increased sleep fragmentation in siblings, potentially indicating a subclinical spectrum. As siblings and patients share much of the genetic and environmental background (same household), siblings provide an optimal comparison group for spectrum analysis. Lastly, we added split-night analyses of stage transitions, dividing the nocturnal PSG into two equal halves, and assessing whether group effects or clinical phenotype associations varied by night-half.

We hypothesize that NT1 patients show greater sleep stage fragmentation and different stage transition probability patterns compared to their non-narcoleptic siblings. Within-group we expect fragmentation to be associated with clinical and biological markers in both patients and siblings. We hypothesize that high-resolution 5-s sleep staging will provide more details on sleep fragmentation, and that sleep fragmentation will be more pronounced in the second half of the night, enhancing the sensitivity to detect group differences and transition patterns that may be concealed by 30-s epoch and/or full-night analyses.

## Materials and Methods

### Cohort

Between February 2015 and July 2024, 154 NT1 patients and 123 siblings were recruited at our Norwegian Centre of Expertise for Neurodevelopmental Disorders and Hypersomnias (NevSom), Oslo University Hospital, Norway. NT1 diagnosis was confirmed in patients and excluded in siblings according to ICSD-3 criteria [63], by European Sleep Research Society (ESRS) certified somnologists and NT1 experts (S.K.H. and B.H.H.). All NT1 patients had disease onset after the 2009 H1N1-vaccination campaign.

All participants (patients and siblings) underwent physical examination and semi-structured interviews based on validated questionnaires addressing narcolepsy and other sleep disorders [64, 65]. Subjective sleepiness was assessed using the Epworth Sleepiness Scale (ESS), where scores ≥11/24 indicated EDS [66]. Participants were interviewed about their lifetime experiences with cataplexy (cataplexy-like episodes in siblings, defined as rare episodes of muscle weakness in response to emotional stimuli), HH, and SP.

Overnight PSG and multiple sleep latency test (MSLT) were conducted in patients and siblings according to AASM standards [31]. Wrist actigraphy was worn for 10–14 days by the majority (87%) of the included participants to evaluate potential circadian disturbances and/or short sleep time. A minority of the cohort had <14 days of actigraphy: 9% (12 patients and 8 siblings) had 2–9 days of actigraphy; 4% (8 patients and 2 siblings) had 0–1 day of actigraphy. These were kept in the analyses as actigraphy was not essential for confirmation or exclusion of narcolepsy (the NT1 diagnosis was, in addition to PSG + MSLT, secured by CSF hypocretin deficiency in 19/20 patients, and by clearcut cataplexy and HLA-DQB1^*^06:02-positivity in the only patient where CSF hcrt-1 was not measured, respectively; for the siblings, narcolepsy was excluded as none had or cataplexy and none had an MSLT mean sleep latency ≤ 8 min and ≥2 SOREMPs; one sibling had an ESS of 15 but did not report feeling sleepier than his peers). CSF hcrt-1 levels were available for 120 NT1 patients. In six patients, who had repeated CSF hcrt-1 measurements, the one closest in time to inclusion was used. The Norwegian CSF hypocretin-1 cutoff is <150 pg/mL [67] and one included NT1 patient (HLA-DQB1^*^06:02-positive, with cataplexy) had two borderline levels (150 pg/mL and 152 pg/mL). All patients and siblings were HLA-typed. H1N1-vaccination (Pandemrix) status of the participants was retrieved from the national registry (SYSVAK). Twelve patients and two siblings not listed as vaccinated in SYSVAK were still classified as vaccinated in our study based on plausible self/parental reports of vaccination at school or their workplace.

Exclusion criteria were technically failed or missing PSG recordings, total sleep time (TST) < 6 h, and apnea-hypopnea index (AHI) > 5. According to these criteria, 24 patients and 19 siblings were excluded. Additionally, two atypical patients were excluded: one patient with initially low CSF hcrt-1 levels and subjective EDS but no objective findings on PSG + MSLT, whose symptoms and hcrt-1 levels later normalized; one patient with initial subjective EDS, reports of cataplexy and a positive MSLT (done locally), but normal CSF hcrt-1 levels, where subsequently symptoms became atypical and PSG + MSLT normalized. One sibling with reports of subjective EDS and possible cataplexy had a positive MSLT (sleep latency < 8 min; 3/5 SOREMPs) but also signs of sleep deprivation/circadian misalignment on actigraphy, hence she was excluded as it was not possible to group her. This resulted in a total inclusion of 125 patients and 100 siblings (Table 1).

**Table 1:** Demographics, clinical features, and symptoms in NT1 patients and non-narcoleptic siblings

	**Patients (*N* = 125)**	**Siblings (*N* = 100)**	***P*-value**
**Demographics**			
Sex (females)	79 (63.2%)	55 (55%)	.222
Age at inclusion (years)	21.6 ± 10.8	21.6 ± 11.4	.905
**Clinical features**			
Age at disease onset (years)	15.5 ± 10.1		
Disease duration (years)	6.2 ± 2.4		
HLA-DQB1^*^06:02 positive (yes)	125 (100%)	61 (61%)	<.0001^*^
H1N1-vaccinated (yes)	107 (85.6%)	73 (73%)	.015^*^
Undetectable CSF hcrt-1 levels (<40 pg/mL) (yes)^†^	71/120 (59.2%)		
Low but detectable CSF hcrt-1 levels (40–150 pg/mL) (yes)^†^	49/120 (40.8%)		
**Symptoms**			
ESS score^‡^	17.7 ± 3.9 (*n* = 123)	5.1 ± 4.0 (n = 99)	<.0001
Cataplexy (-like)^§^	120 (96%)	15 (15%)	<.0001^*^
HH^||^	105 (85%)	17/99 (17.2%)	<.0001^*^
SP^||^	91 (72.8%)	15/99 (15.2%)	<.0001^*^
EDS, cataplexy (-like), HH, and SP^||^	73 (58.4%)	1/99 (1%)	<.0001^*^
EDS, cataplexy (-like), HH, or SP^||^	125 (100%)	35/99 (35.4%)	<.0001^*^

Values are presented as mean ± standard deviation. Binary variables are presented as counts. The *p*-values are from linear mixed-effects models adjusted for age and sex; however, *p*-values marked with ^*^are from Fisher’s exact test. HH = hypnagogic hallucinations; SP = sleep paralysis; ESS = Epworth Sleepiness Scale; EDS = Excessive daytime sleepiness defined as ESS > 10; CSF hcrt-1 = cerebrospinal fluid hypocretin-1. The cohort includes one pair of monozygotic female twins and one pair of dizygotic twins discordant for NT1.

^†^CSF hcrt-1 was only measured in patients (except five patients), and low levels are defined as 40–150 pg/mL (below one-third of the CSF hcrt-1 levels in the general Norwegian population), whereas undetectable levels are defined as <40 pg/mL.

^‡^ESS scores were missing for two patients and one sibling.

§ Cataplexy-like symptoms in siblings were defined as lifetime-experience of emotion-triggered muscle weakness.

|| HH and SP information was missing for one sibling.

Patients were required to pause narcolepsy medication (stimulants, antidepressants, sodium oxybate) for 14 days prior to PSG and MSLT. One patient only paused modafinil for nine days, and one patient paused venlafaxine for seven days but were included. Four patients and seven siblings continued other medication where sleepiness or somnolence is listed as a potential side effect in 1–10 per cent (loratadine, desloratadine, cetirizine, lamotrigine, mesalamine, ranitidine, and montelukast), but of these the included siblings reported not feeling sleepier than others in their age group.

The study was approved by the Norwegian South-East Regional Committees for Medical and Health Research Ethics (REK, ref. 2014/450). All participants provided written informed consent to be included in the study, with parents giving consent on behalf of their children <16 years.

### Sleep stage scoring

The sleep recordings were done using the SOMNOmedics plus system (Domino software, version 2.9.0, SOMNOmedics, Randersacker, Germany). The PSG recordings were sleep stage scored in 30-s epochs by our ESRS certified somnologist-technician (J.V.) following the AASM guidelines [31]. Additionally, automatic sleep stage scoring was done in 5-s mini-epochs using an optimized version of U-Sleep, an automatic deep-learning sleep stage classifier with high performance in epochs originally developed by Perslev et al. [38], which we in our previous study optimized to a similarly high performance in mini-epochs by re-training the original U-Sleep model against J.V.’s 5-s mini-epoch scorings via transfer learning [39].

The optimized U-Sleep model was in the present study applied to score 5-s mini-epochs using electroencephalography (EEG) channels C3:A2, C4:A1, F3:A2, F4:A1, O1:A2, and O2:A1, as well as EOG1:A2 and EOG2:A1. The original U-Sleep classifier code [38] was obtained via a link provided in the original article (https://github.com/perslev/U-Time, accessed March 29, 2023), and pre-trained weights were kindly provided by the first author (M.P.), who also contributed to and co-authored our subsequent U-Sleep re-training publication [39]. More details of the original U-Sleep model can be found in Perslev et al.’s original publication [38], and our U-Sleep transfer learning approach can be found in our recent publication [39].

### Transition indices, probabilities, and wake-periods

The sleep stage transition analyses were performed in the period between lights off and lights on. Transition indices were calculated as number of transitions between all stages, between non-REM (NREM) and REM sleep, and between sleep (NREM1 (N1), NREM2 (N2), NREM3 (N3), REM) and wake divided by TST for both 30-s epochs and 5-s mini-epochs.

To further examine stage transitions, we applied a discrete-time Markov chain across the five sleep stages (N1, N2, N3, REM, wake) in both epochs and mini-epochs. The Markov model assumes that the probability of transitioning from one sleep stage to another depends only on the originating stage.

For each participant, a transition probability matrix was generated by counting all stage-to-stage transitions and normalizing these by the total number of transitions from the originating stage. Diagonal elements represent the probability of remaining in the same stage (i.e. stability), while off-diagonal elements reflect the likelihood of switching to other stages. For example, if a participant remained in N2 for 390 out of 432 transitions, the probability of staying in N2 would be 0.90 (90%).

To further explore if a more detailed analysis of brief versus longer wake-periods reflects different aspects of sleep–wake instability and potentially provide specific biomarkers, we examined the dynamics of wake-periods of varying durations. Our main rationale for applying 5-s mini-epoch scorings was to obtain a more temporally precise and biologically representative sleep staging of all five sleep stages (NREM 1–3, REM, and wake). Hence, due to the known sleep–wake instability (disturbed nighttime sleep) in NT1 [3, 4], we utilized the mini-epoch concept to perform additional analyses of stage wake as determined by the automatic scorings of mini-epochs. We divided wake-periods into three sub-categories based on their duration in the mini-epoch scorings: short wake-periods (one mini-epoch; ≤5 s), intermediate wake-periods (two-three mini-epochs; 5–15 s), and long wake-periods (>three mini-epochs; >15 s) and counted the numbers in each category per TST. These sub-categories were further analyzed in split-night models to assess potential differences across night halves.

### Statistics

We used Fisher’s exact test to compare binary variables. Continuous variables were reported as the mean ± standard deviations and group differences were reported as *β*-estimates with 95% confidence intervals. To analyze group differences, we used the linear mixed-effects model (LME) using the built-in function *fitlme* in MATLAB 2023b. This model accounts for family-relatedness in the data by including a random effect (family-ID) to capture within-family variation in our case caused by the patient/sibling relatedness. For transition probability differences, the LME model was applied per matrix-cell adjusting for sex, age, and family-relatedness.

The LME model was also used to analyze transition indices within the siblings, including relevant clinical predictors (H1N1-vaccination, symptoms, HLA-DQB1^*^06:02) adjusting for age, sex, and family-relatedness. In the full-night patient group analysis, multivariate linear regression was used (as the patients were not related to each other except for two narcoleptic half-brothers), including clinical predictors (H1N1-vaccination, symptoms, CSF hcrt-1 levels) adjusting for age, sex, and disease duration. Participants with missing values (CSF hcrt-1 levels, HH, SP, or ESS) were excluded only from analyses in which the respective variable served as a predictor.

To assess potential changes in sleep fragmentation across the night, we divided the total sleep period into two equal halves (first and second night-half). Each participant thus contributed one value per night-half per transition index/wake-period. We then compared transition indices and wake-periods between night-halves using LME models with night-half and clinical predictors (e.g. H1N1-vaccination, symptoms, CSF hcrt-1 levels, HLA-DQB1^*^06:02) as fixed effects and participant-ID as a random effect to account for repeated measures.

To evaluate whether the sleep fragmentation differed by night-half in patients compared to siblings, we assessed the interactions between night-half effect and group effect including post hoc analyses. We also did post hoc analyses for significant associations when accounting for night-half in the analyses of patient and siblings separately. An overview of all models fitted in current study is provided in Table S1.

For all analyses, we log-transformed dependent variables when visual inspection of residuals via Q–Q plots showed non-normality. Standardized effect sizes (Cohen’s *d*) were computed as the estimated coefficient divided by the model’s residual standard deviation (0.2, 0.5, and 0.8 indicating small, medium, and large effects [68]). To account for multiple comparisons in the transition probability analyses (50 LME models), we applied a Benjamini-Hochberg correction with a false discovery rate of 0.05. Due to the explorative nature of the sub-analyses with clinical predictors (e.g. H1N1-vaccination, symptoms, CSF hcrt-1 levels, HLA-DQB1^*^06:02), we did not apply correction to those analyses; hence significant findings should be interpreted with caution.

## Results

### Demographics for NT1 patients and non-narcoleptic siblings

Table 1 summarizes demographics and clinical features of the NT1 patients and their non-narcoleptic siblings. As seen, the NT1 cohort consisted of typical HLA-DQB1^*^06:02-positive and hypocretin deficient NT1 patients, where the majority additionally had cataplexy and were H1N1-vaccinated with Pandemrix. The groups were comparable regarding age and sex. As expected, due to relatedness, 61% of siblings were also HLA-DQB1^*^06:02-positive and 73% were H1N1-vaccinated with Pandemrix, but siblings had significantly lower ESS and lifetime experience of core narcoleptic symptoms (cataplexy-like episodes, HH, and SP) than patients.

### Sleep characteristics in 30-s epochs and 5-s mini-epochs

PSG sleep features analyzed with both human-scored 30-s epochs and automatically scored 5-s mini-epochs in NT1 patients and siblings are shown in Table 2. The overall sleep architecture found in the NT1 patients in 30-s epochs aligned with well-established nocturnal PSG characteristics of NT1 [30]. Patients had longer time in bed (TIB), lower sleep efficiency, spent more time in wake, N1, and REM sleep, and less time in N2 and N3 sleep compared to their non-narcoleptic siblings. Sleep latency and REM latency were both shorter in patients, and SOREMPs (minimum one epoch of REM sleep <15 min after sleep onset) were present in 77 patients. Siblings had an overall sleep architecture consistent with normal sleep, including higher sleep efficiency and longer sleep and REM latencies, however, two siblings (both males) had SOREMPs. Sub-analyses showed that the siblings’ SOREMPs originated from N2-REM transitions. Neither of the siblings with SOREMPs reported cataplexy-like episodes nor EDS, but both were HLA-DQB1^*^06:02-positive and H1N1-vaccinated.

**Table 2:** Sleep characteristics in NT1 patients and non-narcoleptic siblings based on 30-s epochs and 5-s mini-epochs

**Sleep characteristics**	**30-s Epochs**				**5-s Mini-epochs**			
	**Siblings (*N* = 100)**	**Patients (*N* = 125)**	**Effect size**	***P*-value**	**Siblings (*N* = 100)**	**Patients (*N* = 125)**	**Effect size**	***P*-value**
TIB (hh:mm)	08:40 ± 0:40	09:00 ± 0:51	0.49	.001	08:40 ± 0:40	09:00 ± 0:51	0.49	.001
TST (hh:mm)	08:10 ± 0:44	08:09 ± 0:55	−0.01	.96	07:59 ± 0:46	07:50 ± 0:57	−0.20	.15
SE (%)	94.0 ± 4.4	90.8 ± 6.8	−0.61	<.0001	92.1 ± 4.7	87.3 ± 7.1	−0.94	<.0001
Wake (%)	6.0 ± 4.4	9.3 ± 6.8	0.61	<.0001	7.9 ± 4.7	12.7 ± 7.1	0.93	<.0001
N1 (%)	4.1 ± 2.1	8.6 ± 5.5	1.14	<.0001	4.8 ± 2.8	9.6 ± 5.2	1.21	<.0001
N2 (%)	44.3 ± 7.7	37.7 ± 8.1	−0.93	<.0001	57.4 ± 6.2	49.7 ± 8.4	−1.13	<.0001
N3 (%)	25.2 ± 10.4	20.8 ± 9.5	−0.69	<.0001	15.9 ± 7.4	12.5 ± 6.5	−0.83	<.0001
REM (%)	20.4 ± 4.4	23.6 ± 6.7	0.56	<.0001	14.0 ± 3.9	15.5 ± 4.9	0.39	.0067
Sleep latency (mm:ss)	13:13 ± 12:10	05:30 ± 7:55	−1.19	<.0001	07:04 ± 06:14	4:07 ± 6:35	−0.79	<.0001
REM latency (mm:ss)	94:54 ± 46:49	36:55 ± 65:15	−1.60	<.0001	82:22 ± 54:10	32:00 ± 45:15	−1.04	<.0001
SOREMP (yes)	2	77		<.0001^*^	14^†^	75^†^		<.0001^*^

Values are presented as mean ± standard deviation. Binary variables are presented as counts. The standardized effects sizes (Cohen’s *d*, calculated as estimate divided by the residual standard deviation) and *p*-values are from linear mixed-effects models adjusted for age and sex; *p*-values marked with ^*^are from Fisher’s exact test. The models were adjusted for age and sex. TIB = time in bed, TST = total sleep time, SE = sleep efficiency, N1/N2/N3 = non-rapid eye movement sleep stages 1/2/3, REM = rapid eye movement sleep, SOREMP = sleep-onset REM periods (identified from the PSG). TIB and TST are reported in hours and minutes (hh:mm); latencies are reported in minutes and seconds (mm:ss). Percentages are based on TIB and thus include wake.

^†^In mini-epochs SOREMP corresponds to early-appearing REM periods, i.e. one mini-epoch of REM sleep <15 min after sleep onset.

In 5-s mini-epochs, the sleep stage distributions differed from those found in 30-s epochs. In the mini-epochs, TST and sleep efficiency was lower than in epochs in both groups. Wake, N1, and N2 fractions were higher, N3 and REM fractions were lower, and sleep and REM latencies were shorter in mini-epochs compared to epochs in both groups. The same group differences (patients vs. siblings) for the individual sleep variables remained significant in mini-epochs as in epochs. Furthermore, in mini-epochs, early-appearing REM sleep (minimum one REM mini-epoch <15 min after sleep onset) was present in 75 patients and 14 siblings. In siblings, sub-analyses showed that eight siblings had early-appearing REM mini-epochs originating from wake-REM or N1-REM transitions, and the remaining six siblings had early-appearing REM mini-epochs originating from N2-REM transitions. Ten of the 14 siblings were males. None of the siblings reported cataplexy-like episodes or EDS but 12 were H1N1-vaccinated, and 10 were HLA DQB1^*^06:02-positive.

Standardized effect sizes indicate that when separating NT1 patients from their siblings based on typical sleep characteristics, mini-epochs are better than epochs when looking at TST, sleep efficiency, and percentage and time in stage Wake, N1, N2, and N3. In contrast, epochs separate the groups better when looking at percentage of REM sleep, sleep latency, and REM latency.

### NT1 patients have significantly more sleep-stage transitions than their siblings

**Figure 1:**
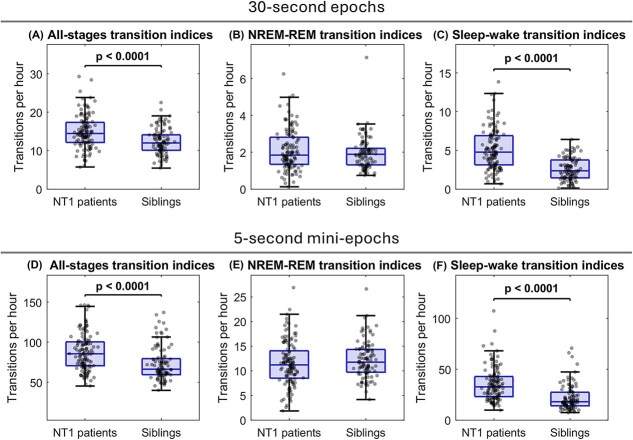
Stage transition indices in NT1 patients and their non-narcoleptic siblings. Boxplots represent group distributions for the different indices across both epochs (30-s) and mini-epochs (5-s). Each dot represents an individual index value. Columns correspond to different indices: All-stages transition index, NREM–REM index, and sleep–wake index. Rows represent the two scoring resolutions: 30-s epochs (top row) and 5-s mini-epochs (bottom row). The mean values are provided in Table S2.

We next examined if we could confirm previous findings of higher NT1 sleep fragmentation in 30-s epochs [48–51] also in our predominantly H1N1-vaccinated NT1 cohort, however here compared to related controls. As shown in Figure 1 and Table S2, in 30-s epochs of full-night PSGs, NT1 patients had significantly higher all-stages transition indices (*p* < .0001, *d* = 0.68) and sleep–wake transition indices (*p* < .0001, *d* = 1.22), but not significantly different NREM–REM transition indices compared to their non-narcoleptic siblings. We then similarly explored if higher resolution sleep staging in 5-s mini-epochs of full-night PSGs would provide more detailed sleep fragmentation assessments in the groups. We found that both patients and siblings had much higher all-stages transition indices, NREM–REM indices, and sleep–wake indices in mini-epochs than in epochs. Similarly to epochs, in mini-epochs, NT1 patients had significantly higher all-stages transition indices (*p* < .0001, *d* = 0.85), higher sleep–wake transition indices (*p* < .0001, *d* = 1.32), and not significantly different NREM–REM indices in full-night PSGs, compared to their siblings.

### Transition probability differences between NT1 patients and siblings

**Figure 2:**
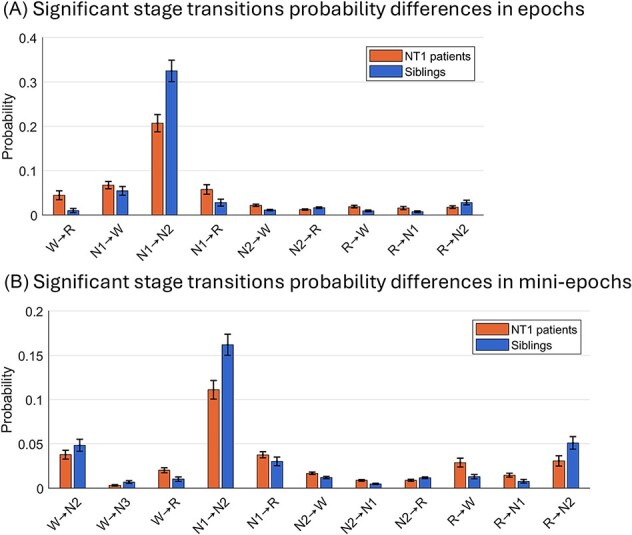
Significant stage transition probability differences between NT1 patients and siblings based on (A) 30-s epochs and (B) 5-s mini-epochs. Bar height shows mean probability across participant and whiskers represent the standard deviation. Each transition probability is normalized by total time in the originating stage to account for group differences in sleep stage distributions; hence, comparisons are only meaningful between transitions originating from the same stage. In epochs, the probability difference in the REM → N3 transition was statistically significant and in mini-epochs differences in the N1 → N3, N3 → N1, N3 → REM, and REM → N3 transitions were statistically significant, but due to their negligible effect sizes these transition probabilities were not included in the figure and not interpreted further. W = stage wake; R = rapid eye movement (REM) sleep.

To further explore transitions between individual sleep stages in full night PSGs of NT1 patients and siblings, we compared transition probabilities between the two groups using LME models, adjusted for age and sex. Figure 2 summarizes the significant group differences (for full transition probability matrices, see Figure S1).

As seen, in epochs (Figure 2, A), NT1 patients were significantly more likely to transition from wake → REM, N1 → REM, REM → wake, REM → N1, N1 → wake, and N2 → wake than siblings, while siblings were more likely to transition from N2 → REM, REM → N2, and N1 → N2, than patients.

In mini-epochs (Figure 2, B), more and new transition patterns emerged. Like in epochs, patients were in mini-epochs significantly more likely than siblings to transition from wake → REM, N1 → REM, REM → wake, REM → N1, and N2 → wake but in mini-epochs additionally also from N2 → N1. Siblings, like in epochs, were in mini-epochs more likely to transition from N2 → REM, REM → N2, and N1 → N2, than patients but in mini-epochs siblings were additionally more likely to transition from wake → N2, and wake → N3.

### Disease severity and H1N1-vaccination is associated with increased sleep fragmentation in NT1

In the full-night PSGs, we explored if sleep fragmentation was associated with disease severity (i.e. having all core narcolepsy symptoms: EDS, cataplexy, HH, SP) or hypocretin deficiency severity (having undetectable [<40 pg/mL] vs. low but detectable [40–150 pg/mL] CSF hcrt-1 levels) within the NT1 group (Table 3). Furthermore, as it has been debated whether H1N1-vaccinated NT1 patients have a different phenotype than sporadic NT1 (some studies report no clinical differences [27, 69–71], while others suggest a more severe or acute presentation in vaccinated patients [21, 28, 29]), we also explored the association between being H1N1-vaccinated and sleep fragmentation.

**Table 3:** Predictors of sleep stage transition indices within the NT1 patient group

	**30-s Epochs**				**5-s Mini-Epochs**			
	No	Yes	Effect size	*P*-value	No	Yes	Effect size	*P*-value
**All-stages transition index**								
H1N1-vaccinated (107/125)	14.5 ± 4.1	15.0 ± 4.3	0.07	.784	84.8 ± 26.1	88.1 ± 22.3	0.23	.384
CSF hcrt-1 undetectable (<40 pg/mL) (71/120)	14.9 ± 3.8	14.8 ± 4.7	−0.03	.873	85.1 ± 20.5	88.8 ± 24.6	0.16	.400
All core symptoms (EDS, cataplexy, HH, SP) (73/125)	15.0 ± 4.2	14.8 ± 4.4	0.04	.851	84.2 ± 20.1	90.0 ± 24.3	0.28	.158
**NREM–REM transition index**								
H1N1-vaccinated (107/125)	2.4 ± 1.3	2.1 ± 1.1	−0.15	.584	11.4 ± 3.6	11.5 ± 4.6	0.05	.847
CSF hcrt-1 undetectable (<40 pg/mL) (71/120)	2.2 ± 1.1	2.0 ± 1.0	−0.14	.449	11.7 ± 5.3	11.2 ± 3.9	−0.10	.597
All core symptoms (EDS, cataplexy, HH, SP) (73/125)	2.2 ± 1.2	2.1 ± 1.0	−0.10	.630	12.3 ± 4.8	10.9 ± 4.2	−0.18	.360
**Sleep–wake transition index**								
H1N1-vaccinated (107/125)	3.9 ± 2.3	5.3 ± 2.6	0.56	.040	34.4 ± 18.6	35.6 ± 15.6	0.29	.280
CSF hcrt-1 undetectable (<40 pg/mL) (71/120)	4.8 ± 2.3	5.3 ± 2.8	0.11	.305	33.0 ± 14.9	36.6 ± 16.8	0.27	.153
All core symptoms (EDS, cataplexy, HH, SP) (73/125)	5.0 ± 2.4	5.3 ± 2.7	0.10	.619	32.5 ± 14.8	37.6 ± 16.6	0.43	.031

Values are presented as mean values ± standard deviation, standardized effects sizes (Cohen’s *d*, calculated as estimate divided by the residual standard deviation) and *p*-values from linear regression models. All models were adjusted for age, sex, and disease duration. CSF hcrt-1 = cerebrospinal fluid hypocretin-1, EDS = excessive daytime sleepiness, HH = hypnagogic hallucinations, SP = sleep paralysis.

**Figure 3:**
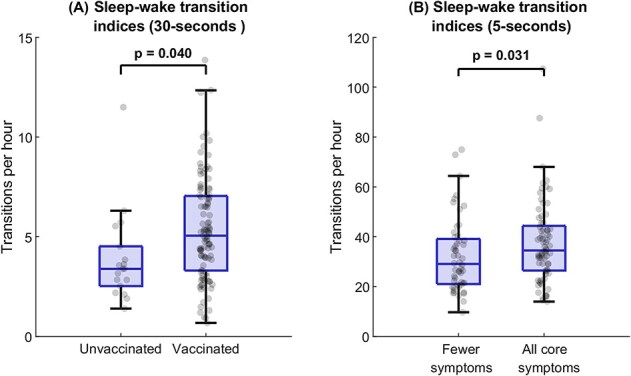
Sleep–wake transition indices stratified by significant predictors within the NT1 patient group. (A) Shows the sleep–wake indices based on 30-s epochs, stratified by H1N1-vaccination status. (B) Shows sleep–wake indices based on 5-s mini-epochs, stratified by presence of the full narcolepsy symptom tetrad. Boxplots show median, interquartile range, and outliers. Points represent individual patient indices. Significant differences are obtained with linear mixed-effect models adjusted for age, sex, and disease duration.

In 30-s epochs, H1N1-vaccinated NT1 patients had higher sleep–wake transition indices than unvaccinated patients (*p* = .040, *d* = 0.56) (Figure 3, A). Neither the patients’ age, sex, nor disease duration had a significant effect on sleep fragmentation in epochs.

In 5-s mini-epochs, patients with all narcolepsy core symptoms (EDS, cataplexy, HH, SP), had higher sleep–wake transition indices (*p* = .031, *d* = 0.43) (Figure 3, B) pointing to that, when analyzing sleep stages more detailed than 30-epochs, sleep fragmentation severity seems to be part of a more severe NT1 sub-phenotype. Similarly, in mini-epochs, longer disease duration was associated with lower all-stages transition indices and sleep–wake transition indices, indicating that sleep fragmentation in NT1 may decrease over time. H1N1-vaccination status was not associated with sleep transitions in mini-epochs. The sex of the patients had no influence, however being older was associated with higher all-stages transition indices and sleep–wake indices in mini-epochs.

### Sleep fragmentation in siblings is not associated with H1N1-vaccination, HLA-DQB1^*^06:02, or core narcolepsy symptoms

As shown in Table 1, even though an ICSD-3 NT1 diagnosis was objectively excluded in all siblings, some of them confirmed that they had experienced one or more of the typical narcolepsy core symptoms (EDS, cataplexy-like episodes, SP, HH). To explore if there was a subclinical narcolepsy spectrum within the sibling group, we analyzed their sleep transition indices in full-night PSGs versus having known NT1 predisposing factors (H1N1-vaccination, HLA-DQB1^*^06:02-positivity) or having a lifetime experience of ≥1 of the core narcolepsy symptoms. However, with both epochs and mini-epochs, there were no significant associations between any of these predictors and the sleep transition indices in the sibling group (Table S3). In both epochs and mini-epochs, the sex of the siblings had no effect, but being older was associated with higher all-stages transition indices and sleep–wake indices.

### Sleep fragmentation varies across the night

To additionally explore if NT1 patients and siblings have different sleep fragmentation patterns in the beginning versus the end of the night, we divided the total sleep period into two halves and compared the transition indices and wake-periods between the first and second night-half.

We performed the split-night analyses using LME models, accounting for family-relatedness, with transition indices and wake-periods (one value per night-half) as dependent variables, adjusted for age and sex, and in models including only patients, additionally for disease duration. The individual participant-ID was additionally included in the models as a random effect to account for repeated measures (two values, i.e. one per night-half, for each participant).

In the 30-s epoch split-night analyses (Table 4), NT1 patients had significantly higher all-stages transition indices than siblings both in the first night-half and second half-night; however, group differences were more pronounced in the second night-half (first half *d* = 0.55, second half *d* = 0.71). Surprisingly, the all-stages transition indices for both patients and siblings were lower in the second half of the night, but a sub analysis showed that in both groups this was primarily driven by more N2/N3 transitions in the first night-half compared to the second night-half. Post hoc analyses showed that while the decrease over night in all-stages transition indices was present in both patients and siblings, it was only significant within the sibling groups. In contrast, when stage transitions were sub analyzed in NREM–REM indices and sleep–wake transition indices, respectively, both these indices increased from first to second half of the night for both patients and siblings. Like in the full-night 30-s epoch models, NREM–REM indices remained comparable between patients and siblings (no significant group differences in both night-halves), and patients had higher sleep–wake transition indices than siblings (in both night-halves).

**Table 4:** Post hoc analyses of night-half effects on transitions indices in NT1 patients and their non-narcoleptic siblings

	**30-s Epochs**			**5-s Mini-epochs**		
	Direction	Effect size	*P*-value	Direction	Effect size	*P*-value
**All-stages transition index**						
Night-half effect in siblings	Lower in 2nd half	−0.41	.003	Lower in 2nd half	−0.90	<.0001
Night-half effect in patients	NS	−0.26	.053	Lower in 2nd half	−0.32	.011
Group-difference in 1st night-half	Higher in patients	0.55	.001	Higher in patients	0.53	.001
Group-difference in 2nd night-half	Higher in patients	0.71	<.0001	Higher in patients	1.11	<.0001
**NREM–REM transition index**						
Night-half effect in siblings	Higher in 2nd half	0.61	<.0001	Higher in 2^nd^ half	0.97	<.0001
Night-half effect in patients	Higher in 2nd half	0.53	<.0001	Higher in 2^nd^ half	0.68	<.0001
Group-difference in 1st night-half	NS	0.16	.360	NS	0.01	.944
Group-difference in 2nd night-half	NS	0.08	.523	Lower in patients	−0.28	.048
**Sleep–wake transition index**						
Night-half effect in siblings	Higher in 2nd half	0.50	.0004	NS	0.19	.211
Night-half effect in patients	Higher in 2nd half	0.43	.001	Higher in 2^nd^ half	0.41	.001
Group-difference in 1st night-half	Higher in patients	1.41	<.0001	Higher in patients	1.47	<.0001
Group-difference in 2nd night-half	Higher in patients	1.42	<.0001	Higher in patients	1.68	<.0001

Post hoc comparisons of night-half effects (i.e. differences between the first and second half of the night) and group differences (NT1 vs. siblings) for three transition indices derived from 30-s epochs and 5-s mini-epochs. Effect sizes are expressed as Cohen’s *d* and *p*-values are obtained from linear mixed-effects models adjusted for age and sex. NT1 = narcolepsy type 1; NS = not significant.

In the 5-s mini-epochs (Table 4), we again found that patients had higher all-stages transition indices than siblings, especially in the second night-half (first half *d* = 0.53, second half *d* = 1.11) and that both groups had higher indices in the first night-half driven by many N2/N3 transitions. Like in epochs, patients and siblings had higher mini-epoch NREM–REM indices in the second night-half. However, the group difference in NREM–REM indices was only significant in the second night-half (patients had significantly lower NREM–REM indices than siblings), while there was no significant group difference in the first night-half. The mini-epoch sleep–wake indices for NT1 patients, as hypothesized, were higher in the second night-half compared to the first half-night, in contrast to their siblings where sleep–wake transition was stable across the night.

Furthermore, as the wake stage in mini-epochs comprised a heterogeneous mix of different wake lengths, we divided wake-periods into three sub-categories: short, intermediate, and long wake-periods (we observed approximately equal amount of the three sub-categories in both patients and siblings; Figure S2). Analyzing these wake sub-categories in mini-epochs (Table 5), we found that patients had significantly more short, intermediate, and long wake-periods than siblings in both night-halves; however, group difference effects were higher in second night-half for all types of wake periods. For both patients and siblings, the number of short wake-periods did not change significantly through the night. Patients, but not siblings, had a significant increase in number of intermediate and long wake-periods in the second night-half, pointing to that NT1 patients have more fragmented sleep in second half of the night. These results indicate that split-night mini-epoch analyses reveal sleep fragmentation patterns and/or differences that are not detectable either in full-night analyses or in epoch analyses.

**Table 5:** Post hoc analyses of night-half effects on wake periods in NT1 patients and their non-narcoleptic siblings

	**Direction**	**Effect size**	***P*-value**
**Short wake periods ≤ 5 s**			
Night-half effect in siblings	NS	−0.10	.474
Night-half effect in patients	NS	0.13	.319
Group-difference in 1st night-half	Higher in patients	1.18	<.0001
Group-difference in 2nd night-half	Higher in patients	1.41	<.0001
**Intermediate wake periods of 5–15 s**			
Night-half effect in siblings	NS	0.14	.320
Night-half effect in patients	Higher in 2^nd^ half	0.30	.017
Group-difference in 1st night-half	Higher in patients	0.93	<.0001
Group-difference in 2nd night-half	Higher in patients	1.10	<.0001
**Long wake periods > 15 s**			
Night-half effect in siblings	NS	0.26	.067
Night-half effect in patients	Higher in 2nd half	0.60	<.0001
Group-difference in 1st night-half	Higher in patients	1.41	<.0001
Group-difference in 2nd night-half	Higher in patients	1.74	<.0001

Post hoc comparisons of night-half effects (i.e. differences between the first and second half of the night) and group differences (NT1 vs. siblings) for wake periods of three different durations derived from 5-s mini-epochs: short (1 mini-epoch, ≤5 s), intermediate (2–3 mini-epochs, 5–15 s), and long (>3 mini-epochs, >15 s). Effect sizes are expressed as Cohen’s *d* and *p*-values are obtained from linear mixed-effects models adjusted for age and sex. NT1 = narcolepsy type 1; NS = not significant.

### Clinical disease severity and hypocretin deficiency severity in NT1 is associated with higher mini-epoch sleep–wake fragmentation—late at night

We lastly explored whether the same predictors used in the full-night models (NT1 core symptom severity, hypocretin deficiency severity, and H1N1-vaccination) were associated with variation in sleep fragmentation across night-halves within the NT1 group, using LME models accounting for repeated measures.

In 30-s epochs, we found no association between any of the predictors and all-stages transition indices, NREM–REM transition indices, sleep–wake transition indices, or wake-periods of any duration, when accounting for night-half (Table S4).

**Figure 4:**
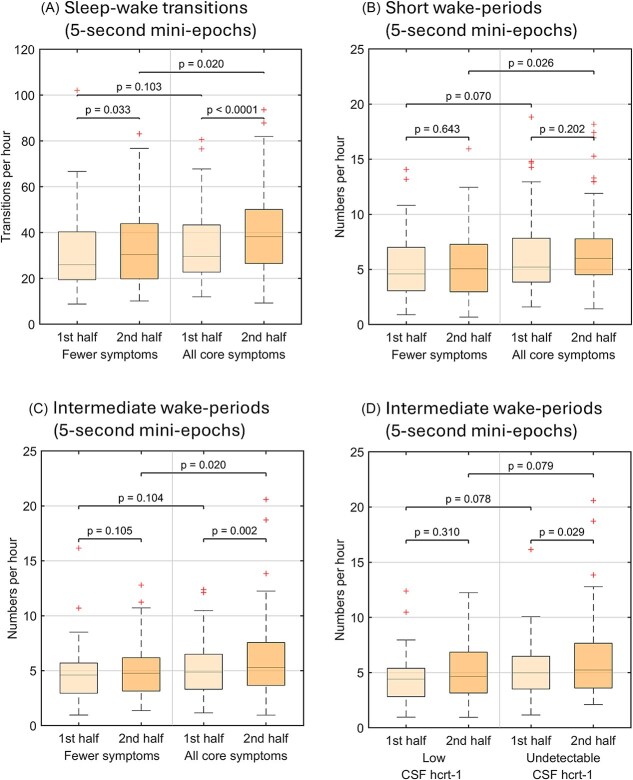
Sleep fragmentation in NT1 patients across night halves stratified by clinical disease severity and CSF hcrt-1 deficiency severity. (A) Sleep–wake transitions from 5-s mini-epochs, number of (B) short wake-periods and (C) intermediate wake-periods, shown by absence/presence of all core symptoms (EDS, cataplexy, HH, SP) across the first and second half of the night. (D) Intermediate wake-periods stratified by CSF hcrt-1 levels (low [40–150 pg/mL] vs. undetectable [<40 pg/mL]) across the first and second half of the night. Boxplots represent median, interquartile range, and outliers. EDS = excessive daytime sleepiness, HH = hypnagogic hallucinations, SP = sleep paralysis.

In 5-s mini-epochs, when including night-half as a fixed effect in our LME models, we found no significant associations between predictors and all-stages transition indices or NREM–REM indices in the patients when accounting for night-half (Table S4). The sleep–wake transition indices were higher in patients with all core NT1 symptoms compared to those with fewer symptoms; however, this group difference was only significant in the second night-half (Figure 4, A). Likewise, patients with all core symptoms had more short and intermediate wake-periods than patients with fewer symptoms, but again, only significant in the second night-half (Figure 4, B and C). Furthermore, NT1 patients with all core narcolepsy symptoms had an increase in numbers of intermediate wake-periods from the first to second night-half, whereas those with fewer symptoms had stable values across the night.

Moreover, there was a trend (not significant) that patients with undetectable CSF hcrt-1 levels (<40 pg/mL) had more intermediate wake-periods in both night-halves than patients with low but detectable levels (40–150 pg/mL) (Figure 4, D). When further analyzing this, patients with undetectable levels had a significant increase in intermediate wake-periods in the second night-half, in contrast to patients with low but detectable levels where numbers of intermediate wake-periods were stable through the night.

Similarly, we performed split-night analyses in the sibling group (Table S5), using the same predictors as in their full-night models. Except that unvaccinated siblings had higher all-stages transition indices than vaccinated siblings in the first night-half, but not in the second night-half (not found in full-night analyses), no other associations between sleep fragmentation and predictors were found in siblings.

## Discussion

In this study, we examined sleep fragmentation in NT1 patients and their non-narcoleptic siblings using both conventional 30-s epochs and high-resolution 5-s mini-epochs. Our results confirm that NT1 patients have significantly more fragmented sleep compared to their siblings, with NT1 sleep–wake instability especially pronounced in the second half of the night. Furthermore, only in mini-epochs, clinical disease severity and hypocretin deficiency severity were associated with more fragmented sleep, and once more, this was more evident when investigating the first and second half of the night separately. In siblings, there were overall no clear associations between clinical symptoms/predictors and sleep fragmentation.

Although our sibling group cannot be considered a true healthy control group, given that first-degree relatives of NT1 patients have an increased risk of developing narcolepsy [59, 60], and several siblings reported lifetime experiences of narcolepsy-like symptoms, it is still informative to contextualize their indices. Based on epochs, our siblings showed averagely 12.4 stage transitions per hour, slightly higher than the 10.1 reported by Laffan et al. [72] in a community-based cohort, but lower than the values of 13.8–14.1 reported by Pizza et al. [48] in patients with non-narcoleptic sleep disorders. However, direct comparisons are limited by methodological or demographical differences in the studies.

Our NT1 patients showed a sleep–wake transition index of 5.2 in 30-s epochs, slightly lower than reported by others [48, 49]. This difference may reflect methodological factors such as variations in cohort demographics, sleep-recording equipment, filter settings, and potential inter-rater variability. Nevertheless, the consistently higher sleep–wake indices across NT1 cohorts, including our predominately H1N1-vaccinated cohort, supports its interpretation as a marker of disease-related sleep–wake instability.

Sorensen et al. [49] found higher NREM–REM indices from epochs in hypocretin deficient NT1 patients (which all patients in our present study are), compared to narcolepsy patients with normal hypocretin levels. In our full-night analysis, we did not find a significant difference in NREM–REM indices between patients and siblings, neither when using 30-s epochs nor 5-s mini-epochs. However, our split-night analyses showed that patients had lower NREM–REM indices than siblings, but only in the second night-half. In concordance, Schoch et al. [33], who in a small study divided epoch into 5-s mini-epochs and manually adapted the scoring when necessary, reported fewer NREM–REM transitions in narcolepsy patients than in controls, although not significantly. The fact that this pattern in our present study was only significant in split-night analyses—and not in full-night analyses at either scoring resolution—indicates that split-night analyses can reveal temporally specific REM-related instability in NT1 that may remain undetectable when averaging across the entire night.

Although transition counts were naturally higher in mini-epochs due to the increased time-resolution, the relative group differences between patients and siblings remained stable. This indicates that mini-epoch scoring does not introduce artificial instability but rather uncovers brief transitions that are missed in coarser scoring. However, considering sleep–wake indices, the group difference was smaller in relative terms in mini-epochs, likely reflecting the sensitivity of mini-epochs to detect brief wake-periods in both groups, elevating the baseline sleep–wake transition indices. Prior work has shown that NT1 patients have markedly more awakenings but only slightly more arousals than controls (in epoch-based studies) [30], supporting this interpretation.

To gain a more detailed understanding of the sleep instability in NT1, we analyzed transition probabilities between the specific sleep stages. These conditional probabilities reflect the likelihood of transitioning to a specific stage, given the current stage, accounting for differences in stage distribution across participants.

In both 30-s epochs and 5-s mini-epochs, NT1 patients showed a distinct pattern of fragmented and abnormal sleep cycling. Both in epochs and mini-epochs, they were more likely than siblings to transition from wake or N1 directly into REM, a pattern also reported by others in epochs [50, 52, 53]. While wake to REM transitions are classically associated with SOREMPs, our finding of their recurrence throughout the night, especially in mini-epochs, suggests ongoing REM instability, possibly associated with brief wake-periods within unstable REM segments. Mini-epochs, which can capture brief wake-periods, are especially sensitive to such instability. In contrast to patients, we found in epochs and mini-epochs that siblings were more likely to transition from N2 to REM, indicating a more stable and well-structured sleep cycle. Siblings were also more likely to follow the normal cycle path from N1 to N2, whereas patients were more likely to transition from N2 to N1, or N1 to wake, suggesting an instability of normal progression from light to deeper sleep. The N2 to N1 transitions were only significantly more likely in patients in 5-s mini-epochs, highlighting how high-resolution scoring can uncover sleep instability that remain hidden in 30-s epochs.

Our findings of transitions out of REM sleep further support the pattern of sleep instability in NT1, as patients were more likely to transition from REM to N1 or wake, and less likely to transition from REM to N2, as also shown by Ferri et al. [73] Unexpectedly, siblings showed higher wake to N3 transition probabilities in mini-epochs, however, these transitions were rare and not observed in epochs. On closer inspection, the wake to N3 transitions were consistently preceded by N2 (i.e. N2–W–N3) rather than sustained wakefulness, suggesting that they reflect brief wake-periods during ongoing sleep segments.

Within the NT1 group, we found that different clinical predictors were associated with altered sleep stage transition indices potentially indicating an underlying disease heterogeneity. Based on epochs, H1N1-vaccination was associated with higher sleep–wake indices, which could point in the direction that H1N1-vaccinated NT1 patients may exhibit more severe symptomatology [21, 28, 29], though other studies have suggested similar phenotypes of pre and post-H1N1 NT1 [27, 69–71, 74]. However, due to a relatively small unvaccinated proportion of our patient cohort (18/125), this comparison was exploratory and underpowered, and the finding should be interpreted with caution.

When analyzing mini-epochs, patients with longer disease duration had lower all-stages transition indices and sleep–wake indices, suggesting a possible partial adaptation or stabilization of sleep architecture over time in NT1. This aligns with studies reporting that some NT1 symptoms, especially EDS and cataplexy, may attenuate or plateau with longer disease duration [30, 75–77].

Patients with all core narcolepsy symptoms had higher sleep–wake indices based on 5-s mini-epochs, suggesting that sleep–wake fragmentation is more pronounced in clinically more affected patients, as also reported by Barateau et al. [57] The narcolepsy core symptoms EDS, cataplexy, HH, and SP have long been a clinically defining symptom tetrad for NT1 [78]; hence, our study supports the proposal that sleep fragmentation constitutes a fifth core NT1 feature including cohorts of H1N1-vaccinated NT1 patients [3, 4]. Notably, this association was only detectable using mini-epochs, i.e. not when using epochs, emphasizing the value of high-resolution sleep staging.

Importantly, epochs and mini-epochs were associated with different clinical predictors, suggesting that the two scoring resolutions may capture different aspects of sleep dynamics. However, the findings should be interpreted differently, as the underlying subgroup compositions differed markedly between the analyses. The H1N1 comparison in 30-s epochs was based on highly unbalanced subgroups (18 vs. 107 patients), making this finding underpowered. In contrast, the core symptom analysis in 5-s mini-epochs was based on more comparable subgroup sizes (52 vs. 73 patients).

We further found that time of night matters when analyzing sleep fragmentation. Patients with all core symptoms had a significant increase in sleep–wake fragmentation both in terms of the sleep–wake index, and also short, and intermediate wake-periods in the second half of the night. This supports our hypothesis that sleep–wake fragmentation is increased across the night in NT1 but more pronounced after the initial sleep pressure has dissipated, and moreover especially evident in the most affected patients.

Likewise, the most hypocretin deficient NT1 patients (i.e. those with undetectable CSF hcrt-1 levels [<40 pg/mL]) had an increase in intermediate wake-periods from first to second half of the night, whereas those with low but detectable levels (40–150 pg/mL) had stable numbers through the night. We also found a non-significant trend of higher frequency of intermediate wake-periods in patients with undetectable CSF hcrt-1 levels in the split-night analysis. Although mini-epoch scoring is a new concept, and we do not know if intermediate wake-periods are comparable to conventional arousals, this finding is potentially in line with a study of a small NT1 sample (25 patients) reporting higher cortical arousal indices in the most hypocretin-deficient patients [79], and another study showing (non-significant) increased sleep–wake transitions in patients with the lowest hypocretin levels [80]; however, they only studied it in full-night analyses. Though not all significant, our hypothesis-generating findings were only detectable with split-night models, suggesting that full-night models overlook biologically meaningful effects that are temporally specific. These findings should be explored further.

Short wake-periods were intended to capture very brief wake intrusions (potentially comparable to short cortical arousals), whereas intermediate wake-periods were intended to capture slightly longer brief awakenings (potentially comparable to longer cortical arousals). Long wake-periods were intended to capture awakenings (that would typically be scored as wake in a 30-s epoch, however, only if located within one epoch, and are therefore only partially comparable to conventional epoch-scored wake). Although the short and intermediate mini-epoch wake-periods may overlap with conventional AASM-defined cortical arousals [31], our aim in applying 5-s mini-epoch scoring was to obtain a more temporally precise and biologically representative sleep stage scoring of all five stages (NREM 1–3, REM, and wake), not to capture or redefine cortical arousals. We did not examine the degree of overlap between wake-periods and cortical arousals in the present study, and future work comparing mini-epoch-derived wake-periods with AASM-defined arousals or awakenings will be required to clarify this.

In mini-epochs, we also identified early-appearing REM periods in several siblings in contrast to only two siblings with SOREMPs in epochs. This finding initially raised questions about potential subclinical features in the siblings as a nocturnal SOREMP in epochs has become a diagnostic criterion for narcolepsy in the recent ICSD-3TR version [81], however, its clinical meaning in mini-epochs remains uncertain. Eight of the siblings transitioned from wake or N1 into REM, which is considered typical for NT1 [82] when assessing sleep in epochs. Notably, the majority of the siblings with early-appearing REM periods were H1N1-vaccinated and HLA-DQB1^*^06:02-positive (known NT1 risk factors), but the absence of EDS and cataplexy 7.8 ± 2.1 years after H1N1-vaccination suggests that further follow-up of these siblings is probably not needed. Importantly, SOREMPs are not specific to narcolepsy and can also occur due to sleep deprivation, circadian misalignment, or shift work [83]. However, it has previously been reported in the general population, that HLA-DQB1^*^02:06 is associated with SOREMPs in males, though in the MSLT and in epochs [84]. Our mini-epoch approach is new; hence, it is unknown if early-appearing REM mini-epochs have clinical/diagnostic implications or not. Moreover, as we did not include a healthy control group, we cannot determine whether such early REM phenomena in mini-epochs also occur in the general population. These findings should be replicated in independent datasets including healthy controls before any interpretation can be made.

We acknowledge that our study has limitations. First, we did not include an unrelated healthy control group. Instead, we used siblings as controls, who reduce environmental and familial confounding but cannot be considered completely healthy controls. This means that, based on present study, we cannot draw conclusions about normality. High-resolution scoring introduces challenges, as mini-epochs can be more difficult to classify consistently, e.g. during biological sleep stage transitions, where the background electroencephalogram (EEG) activity is often ambiguous and contains overlapping sleep features.

In the siblings with early-appearing REM mini-epochs, 4 of 14 siblings transitioned directly from wake to REM, and eye movements in REM and wake are known to be difficult to differentiate. However, three of those four siblings were HLA-DQB1^*^06:02-positive, similar to 70% of the remaining siblings with early-appearing REM mini-epochs, suggesting that this could reflect a true biological phenomenon (i.e. potentially equivalent to the known association between SOREMPs and the HLA-DQB1^*^06:02 allele [84]). However, current diagnostic SOREMP criteria were developed for epochs and may not translate directly to high-resolution scoring, i.e. the diagnostic implication is unknown, and the finding should therefore be interpreted cautiously and replicated in future studies.

Lastly, in wake sub-analyses, we defined durations of short, intermediate, and long wake-periods based on numbers of mini-epochs. These durations were not physiologically validated and may require future refinement.

In conclusion, this study demonstrated that NT1 is associated with pronounced sleep fragmentation, especially sleep–wake fragmentation in the second half of the night. Across several analyses, the use of mini-epochs provided greater insight into group differences and clinically relevant patterns that remained undetected with conventional epochs. This suggests that increased temporal resolution offers not just methodological refinement but meaningful clinical insight. In 5-s mini-epochs, sleep–wake fragmentation was particularly increased in NT1 patients with severe clinical disease and severe hypocretin deficiency (<40 pg/mL), especially in the second half of the night. Our findings indicate clinical relevance of sleep stage transitions analyzed at high resolution as markers of disease severity and heterogeneity and highlight the value of temporally sensitive methods.

## Supplementary Material

Supplementary_materials_zsag015

## Data Availability

The data used in this article cannot be shared publicly since we do not have ethical approval to share the data due to privacy of individuals of the study.
