# Supplementary material for: High-resolution sleep fragmentation assessment in narcolepsy type 1 and their non-narcoleptic siblings: a 5-s mini-epoch study
Source: Sleep. 2026 Jan 28;49(5):zsag015. doi: 10.1093/sleep/zsag015 (PMC13163166; doi:10.1093/sleep/zsag015)
Supplement: Supplementary_materials_zsag015 [file supplementary_materials_zsag015.docx]

High-resolution sleep fragmentation assessment in narcolepsy type 1 and their non-narcoleptic siblings: a 5-second mini-epoch study

Louise Frøstrup Follin^1,2,^*, Rannveig Viste^1^, Janita Vevelstad^1^, Kristin Langdalen^1,2^, Berit Hjelde Hansen^1^, Ragnhild Kristine Berling Grande^1^, Tobias Kaufmann^3,4,5^, Julie Anja Engelhard Christensen^1,6,^, Alexander Neergaard Zahid^7,8^, Marte Kathrine Viken^9,10^, Hilde T. Juvodden^1^, Stine Knudsen-Heier^1,2^

^1^Norwegian Centre of Expertise for Neurodevelopmental Disorders and Hypersomnias (NevSom), Department of Rare Disorders, Oslo University Hospital, Norway

^2^Institute of Clinical Medicine, University of Oslo, Norway

^3^Centre for Precision Psychiatry, Institute of Clinical Medicine, University of Oslo, Norway

^4^Department of Psychiatry and Psychotherapy, University of Tübingen, Germany

^5^German Center for Mental Health (DZPG), Partner site Tübingen, Tübingen, Germany

^6^Novo Nordisk, Hillerød, Denmark

^7^WS Audiology, Lynge, Denmark

^8^Department of Applied Mathematics and Computer Science, Technical University of Denmark, Kgs. Lyngby, Denmark

^9^Department of Immunology, University of Oslo and Oslo University Hospital, Norway

^10^Department of Medical Genetics, University of Oslo and Oslo University Hospital, Norway

*Corresponding author: Louise Frøstrup Follin, Norwegian Centre of Expertise for Neurodevelopmental Disorders and Hypersomnias (NevSom), Oslo University Hospital, Box 4956 Nydalen, 0424 Oslo, Norway. Email: [lofroe@ous-hf.no](mailto:lofroe@ous-hf.no)

# Supplementary Materials

**Figure S1:** Stage transition probability differences between NT1 patients and siblings

**Figure S2:** Mean wake period number

**Table S1:** Statistical models

**Table S2:** Sleep fragmentation indices in NT1 patients and non-narcoleptic siblings

**Table S3:** Sleep stage transition indices with the non-narcoleptic sibling group

**Table S4:** Effects of night-half and predictors on transitions indices in NT1 patients

**Table S5:** Effects of night-half and predictors on transitions indices in siblings


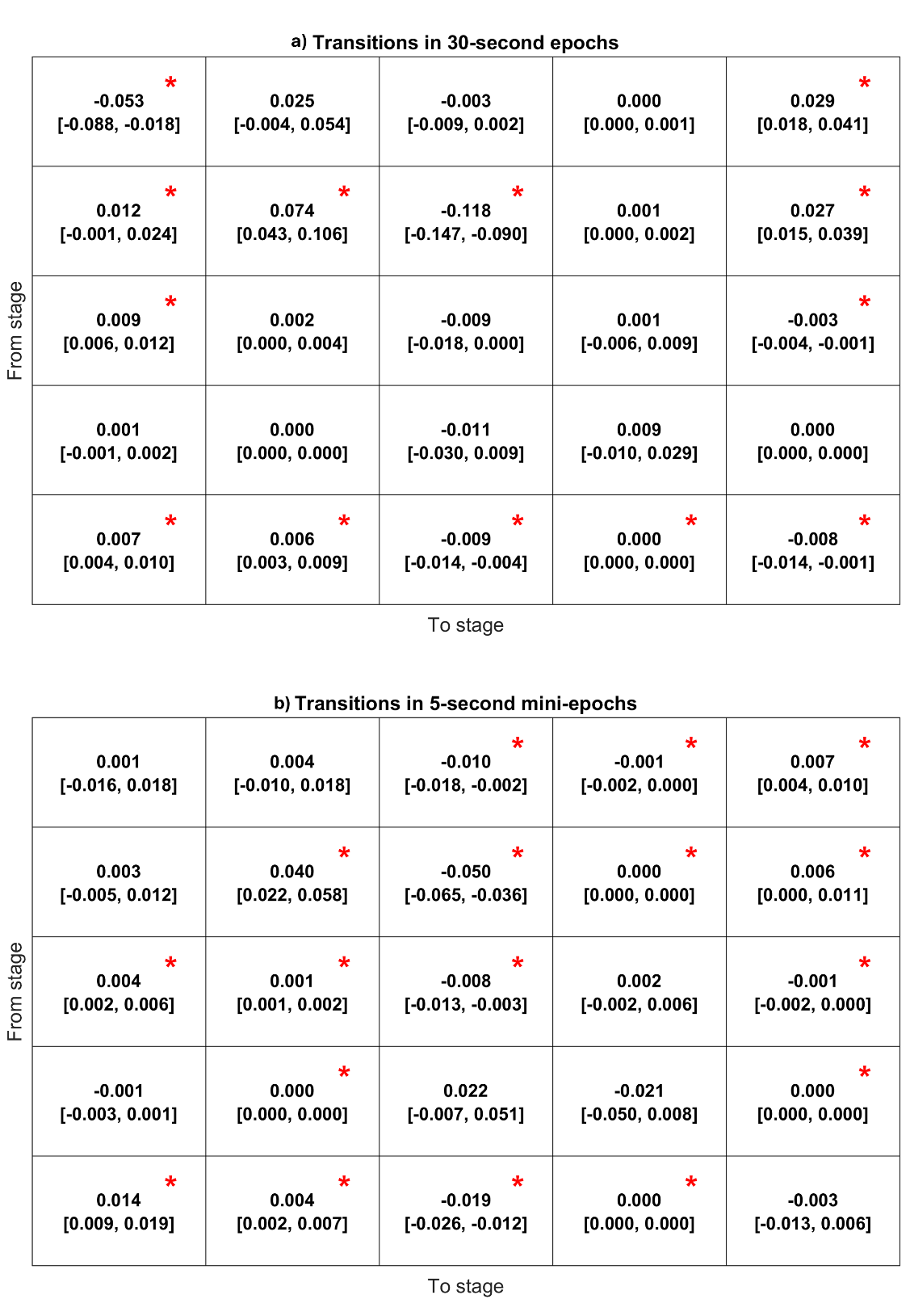

Figure S1: Stage transition probability differences between NT1 patients and non-narcoleptic siblings based on a) epochs and b) mini-epochs. Each matrix cell shows the estimated difference in transition probability and 95% confidence interval (sibling is reference), derived from linear mixed-effects models with one model per cell adjusting for age, sex, and family-relatedness. A red asterisk marks statistically significant differences at *p* < 0.0146 after Benjamini–Hochberg correction.


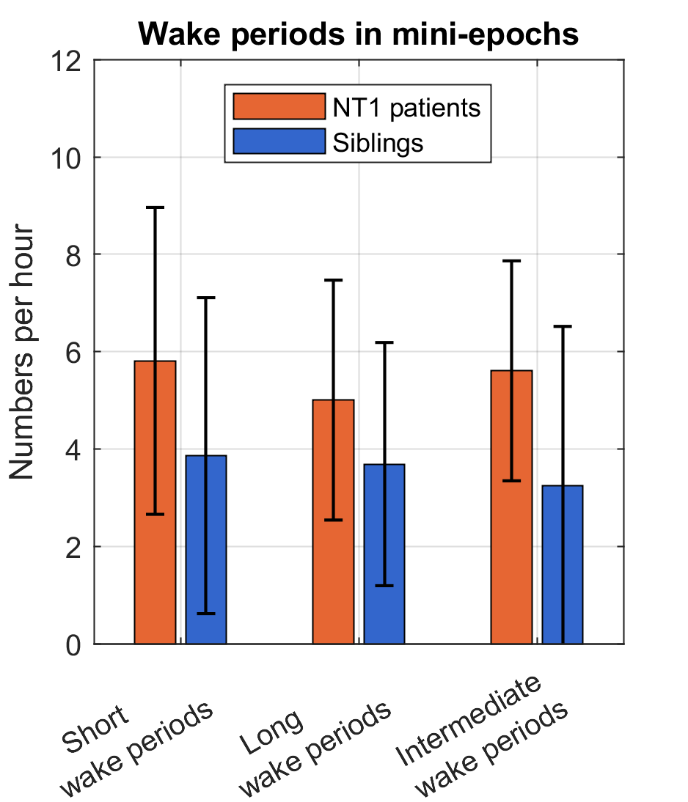


Figure S2: Means ± standard deviations of wake periods of different durations; short (1 wake mini-epoch), intermediate (2–3 wake mini-epochs), and long (>3 wake mini-epochs) per hour of total sleep time, based on 5-second mini-epoch scoring. Bar height shows mean across participant and whiskers represent the standard deviation.

**Table S1: Summary of statistical models**

| Analysis | Resolution | Dependent variables | Method | Models (N) | Predictors | Covariates | Night segmentation |
| --- | --- | --- | --- | --- | --- | --- | --- |
| Group differences (patient/sibling) | 30-seconds | Transition indices | LME | 3 | Group (pat/sib) | age, sex | Full-night |
| Group differences (patient/sibling) | 5-seconds | Transition indices | LME | 3 | Group (pat/sib) | age, sex | Full-night |
| Group differences (patient/sibling) | 30-seconds | Transition indices | LME | 3 | Group (pat/sib), night-half * | age, sex | Split-night |
| Group differences (patient/sibling) | 5-seconds | Transition indices, wake periods | LME | 6 | Group (pat/sib), night-half * | age, sex | Split-night |
| Clinical predictors (patient) | 30-seconds | Transition indices | LR | 3 | H1N1-vaccination CSF hcrt-1 levels  All core symptoms | age, sex, disease duration | Full-night |
| Clinical predictors (patient) | 5-seconds | Transition indices | LR | 3 | H1N1-vaccination CSF hcrt-1 levels  All core symptoms | age, sex, disease duration | Full-night |
| Clinical predictors (patient) | 30-seconds | Transition indices | LME | 3 | H1N1-vaccination CSF hcrt-1 levels  All core symptoms Night-half | age, sex, disease duration | Split-night |
| Clinical predictors (patient) | 5-seconds | Transition indices, wake periods | LME | 6 | H1N1-vaccination CSF hcrt-1 levels  All core symptoms Night-half | age, sex, disease duration | Split-night |
| Clinical predictors (sibling) | 30-seconds | Transition indices | LME | 3 | H1N1-vaccination HLA-DQB1*06:02  ≥1 core symptom | age, sex | Full-night |
| Clinical predictors (sibling) | 5-seconds | Transition indices | LME | 3 | H1N1-vaccination HLA-DQB1*06:02  ≥1 core symptom | age, sex | Full-night |
| Clinical predictors (sibling) | 30-seconds | Transition indices | LME | 3 | H1N1-vaccination HLA-DQB1*06:02  ≥1 core symptom Night-half | age, sex | Split-night |
| Clinical predictors (sibling) | 5-seconds | Transition indices, wake periods | LME | 6 | H1N1-vaccination HLA-DQB1*06:02  ≥1 core symptom Night-half | age, sex | Split-night |
| Markov analysis, group difference (patient/sibling) | 30-seconds | Transition probabilities | LME | 50 | Group (pat/sib) | age, sex | Full-night |
| Markov analysis, group difference (patient/sibling) | 5-seconds | Transition probabilities | LME | 50 | Group (pat/sib) | age, sex | Full-night |

Summary of linear regression (LR) and linear mixed-effect (LME) model specifications with dependent variables, predictors, and covariates. All LME models included family-ID as a random effect to account family-relatedness. All split-night models included participant-ID as a random effect to account for multiple measurements per participant. *The models included the interaction between group x night-half.

**Table S2: Sleep fragmentation indices in NT1 patients and non-narcoleptic siblings**

|  | Siblings (N=100) | Patients (N=125) | Effect size | p-value |
| --- | --- | --- | --- | --- |
| All-stages transition index (30-seconds) | 12.4 ± 3.4 | 14.9 ± 4.3 | 0.68 | <0.0001 |
| All-stages transition index (5-seconds) | 73.0 ± 20.7 | 87.6 ± 22.8 | 0.85 | <0.0001 |
| NREM-REM index (30-seconds) | 1.9 ± 0.9 | 2.1 ± 1.1 | 0.07 | 0.632 |
| NREM-REM index (5-seconds) | 12.2 ± 3.7 | 11.5 ± 4.5 | -0.26 | 0.207 |
| Sleep-wake index (30-seconds) | 2.6 ± 1.4 | 5.1 ± 2.6 | 1.22 | <0.0001 |
| Sleep-wake index (5-seconds) | 22.0 ± 12.4 | 35.4 ± 16.0 | 1.32 | <0.0001 |

Values are presented as mean ± standard deviation. The effect sizes (Cohen’s d) and p-values are from linear mixed-effects models adjusted for age and sex.

**Table S3: Sleep stage transition indices with the non-narcoleptic sibling group**

|  | **30-second epochs** | | | | **5-second mini-epochs** | | | |
| --- | --- | --- | --- | --- | --- | --- | --- | --- |
|  | All-stages transition index | | | | | | | |
|  | No | Yes | Effect size | p-value | No | Yes | Effect size | p-value |
| H1N1-vaccinated (73/100) | 12.2 ± 3.0 | 12.5 ± 3.5 | 0.63 | 0.062 | 84.5 ± 27.3 | 68.7 ± 15.9 | -0.43 | 0.201 |
| HLA-DQB1*06:02 positive (61/100) | 12.2 ± 3.3 | 12.6 ± 3.4 | 0.17 | 0.547 | 69.8 ± 19.9 | 75.1 ± 21.3 | 0.20 | 0.480 |
| EDS, cataplexy, HH, or SP (35/99) | 12.5 ± 3.4 | 12.3 ± 3.4 | -0.27 | 0.349 | 71.2 ± 19.1 | 76.2 ± 23.4 | 0.03 | 0.921 |
|  | NREM-REM transitions index | | | | | | | |
|  | No | Yes | Effect size | p-value | No | Yes | Effect size | p-value |
| H1N1-vaccinated (73/100) | 1.7 ± 0.6 | 2.0 ± 0.9 | 0.25 | 0.383 | 11.8 ± 4.4 | 12.4 ± 3.5 | 0.04 | 0.886 |
| HLA-DQB1*06:02 positive (61/100) | 2.0 ± 0.7 | 1.9 ± 1.0 | -0.13 | 0.598 | 11.8 ± 3.7 | 12.4 ± 3.8 | 0.17 | 0.410 |
| EDS, cataplexy, HH, or SP (35/99) | 1.9 ± 0.9 | 1.9 ± 0.7 | 0.12 | 0.626 | 12.6 ± 3.9 | 11.5 ± 3.2 | -0.23 | 0.292 |
|  | Sleep-wake transitions index | | | | | | | |
|  | No | Yes | Effect size | p-value | No | Yes | Effect size | p-value |
| H1N1-vaccinated (73/100) | 3.1 ± 1.4 | 2.4 ± 1.4 | 0.02 | 0.940 | 28.4 ± 15.6 | 19.6 ± 10.1 | -0.17 | 0.529 |
| HLA-DQB1*06:02 positive (61/100) | 2.5 ± 1.3 | 2.7 ± 1.5 | 0.02 | 0.943 | 21.1 ± 10.8 | 22.7 ± 13.4 | 0.00 | 0.992 |
| EDS, cataplexy, HH, or SP (35/99) | 2.5 ± 1.4 | 2.8 ± 1.3 | 0.27 | 0.269 | 21.0 ± 11.4 | 23.7 ± 14.1 | 0.15 | 0.523 |

Values are presented as mean values ± standard deviation, standardized effects sizes (Cohen’s d, calculated as estimate divided by the residual standard deviation) and p-values from fully adjusted linear mixed-effects models. All models were adjusted for age and sex. EDS = excessive daytime sleepiness, HH = hypnagogic hallucinations, SP = sleep paralysis.

**Table S4: Effects of night-half and predictors on transitions indices in NT1 patients**

| Index (resolution) | H1N1-vaccination | CSF hcrt-1 undetectable (<40 pg/ml) | All core symptoms (EDS, cataplexy, HH, SP) |
| --- | --- | --- | --- |
| All-stages transition index  (epochs) | NS | NS | NS |
| All-stages transition index  (mini-epochs) | NS | NS | NS |
| NREM-REM index (epochs) | NS | NS | NS |
| NREM-REM index (mini-epochs) | NS | NS | NS |
| Sleep-wake index (epochs) | NS | NS | NS |
| Sleep-wake index (mini-epochs) | NS | NS | Night-half effect in patients with <4 symptoms:  d=0.43, p=0.033 Night-half effect in patients with all symptoms:  d=0.65, p<0.0001 Group difference in 1^st^ half:  d=0.51, p=0.103 Group difference in 2^nd^ half:  d=0.74, p=0.020 |
| Short wake-periods (mini-epochs) | NS | NS | Night-half effect in patients with <4 symptoms:  d=0.09, p=0.643 Night-half effect in patients with all symptoms:  d=0.22, p=0.202 Group difference in 1^st^ half:  d=0.54, p=0.070 Group difference in 2^nd^ half:  d=0.66, p=0.026 |
| Intermediate-wake (mini-epochs) | NS | Night-half effect in patients with low CSF:  d=0.44, p=0.310 Night-half effect in patients with undetectable CSF:  d= 0.44, p=0.029 Group difference in 1^st^ half:  d= 0.49, p=0.078 Group difference in 2^nd^ half:  d=0.49, p=0.079 | Night-half effect in patients with <4 symptoms:  d=0.33, p=0.105 Night-half effect in patients with all symptoms:  d=0.53, p=0.002 Group difference in 1^st^ half:  d=0.47, p=0.104 Group difference in 2^nd^ half:  d=0.67, p=0.020 |
| Long wake-periods (mini-epochs) | NS | NS | NS |

Night-half effects and group differences in NT1 patients when including the predictors: H1N1-vaccination, low or undetectable CSF hcrt-1 levels, and having all core narcolepsy symptoms (EDS, cataplexy, HH, SP) for stage transition indices and wake periods based on epochs (30-seconds) and mini-epochs (5-seconds). Reported standardized effect sizes (Cohen’s d) and p-values are from linear mixed-effects models adjusted for age, sex, and disease duration. Post hoc analyses were conducted for models in which either the interaction term or the predictor showed a significant or near-significant effect (p<0.1). Models labeled NS indicate no significant/near-significant effects of either the predictor or the interaction term. NT1 = narcolepsy type 1, NS = not significant, CSF hcrt-1 = cerebrospinal fluid hypocretin-1, EDS = excessive daytime sleepiness, HH = hypnagogic hallucinations, SP = sleep paralysis.

**Table S5: Effects of night-half and predictors on transitions indices in non-narcoleptic siblings**

| Dependent variable (resolution) | H1N1-vaccination | HLA-DQB1*06:02 positive | EDS, cataplexy, HH, or SP |
| --- | --- | --- | --- |
| All-stages transition index  (epochs) | Night-half effect in unvaccinated siblings:  d=0.43, p<0.0001 (lower in 2^nd^ half) Night-half effect in vaccinated siblings:  d=0.65, p<0.0001 (lower in 2^nd^ half) Group difference in 1^st^ half:  d=0.51, p=0.042 (lower in vaccinated) Group difference in 2^nd^ half:  d=0.74, p=0.450 | NS | NS |
| All-stages transition index  (mini-epochs) | NS | NS | NS |
| NREM-REM index (epochs) | NS | NS | NS |
| NREM-REM index (mini-epochs) | NS | NS | NS |
| Sleep-wake index (epochs) | NS | NS | NS |
| Sleep-wake index (mini-epochs) | NS | NS | NS |
| Short wake-periods (mini-epochs) | NS | NS | NS |
| Intermediate-wake (mini-epochs) | NS | NS | NS |
| Long wake-periods (mini-epochs) | NS | NS | NS |

Night-half effects and group differences in siblings when including the predictors: H1N1-vaccination, HLA-DQB1*06:02-status, or narcolepsy-like symptom for stage transition indices and wake periods in epochs (30-seconds) and mini-epochs (5-seconds). Reported standardized effect sizes (Cohen’s d) and p-values are from linear mixed-effects models adjusted for age and sex. Post hoc analyses were conducted for models in which either the interaction term or the predictor showed a significant or near-significant effect (p<0.1). Models labeled NS indicate no significant/near-significant effects of either the predictor or the interaction term. NS = not significant, EDS = excessive daytime sleepiness, HH = hypnagogic hallucinations, SP = sleep paralysis.
